# Impact of Home Parenting Environment on Cognitive and Psychomotor Development in Children Under 5 Years Old: A Meta-Analysis

**DOI:** 10.3389/fped.2021.658094

**Published:** 2021-09-28

**Authors:** Qing Yang, Jinlian Yang, Liming Zheng, Wei Song, Lilan Yi

**Affiliations:** ^1^Department of Pediatrics, The First Affiliated Hospital of Hunan University of Medicine, Huaihua, China; ^2^Nursing College, Hunan University of Medicine, Huaihua, China; ^3^Department of Child Health Care, Huaihua Maternal and Child Health Hospital, Huaihua, China

**Keywords:** home parenting environment, children, cognitive development, psychomotor development, meta-analysis

## Abstract

This study aims to evaluate the relationship between home parenting environment and the cognitive and psychomotor development in children under 5 years old by using meta-analysis. A systematic search of the Chinese and English databases including Pubmed, Embase, the Cochrane Library, CNKI, Weipu, Wanfang, and CBMdisc databases from January 1, 1990, to July 31, 2021, was performed. Articles concerning the relationship between home parenting environment and the cognitive and psychomotor development in children under 5 years old were included. Review Manager 5.4 was used for meta-analysis. Subgroup analysis in terms of age and region were performed. A total of 12 articles were included, including 11 in English and 1 in Chinese. Meta-analysis showed that there was significant relationship between home parenting environment and the cognitive and psychomotor development of children (*r* = 0.31; *r* = 0.21). Subgroup analysis showed that correlation between home parenting environment and the cognitive and psychomotor development of children was stronger in children over 18 months compared to those under 17 months [(*r* = 0.33, *r* = 0.21) vs. (*r* = 0.28, *r* = 0.17)]. The converted summary *r* value between home parenting environment and cognitive development in developing and developed countries was both 0.32. Conclusively, there is a positive correlation between the home parenting environment and the cognitive and psychomotor development of children under 5 years old. Improving the home parenting environment of children is beneficial to promote their early development.

## Introduction

Studies (1, 2) have shown that the number of children under 5 years of age who have development deficiency due to poverty, disease, neglect of care and other reasons in developing countries worldwide has increased from 200 million to 250 million from 2007 to 2017, which includes 17.1 million in China. According to the 2020 Global Nutrition Report on malnutrition, nearly one in every four children under 5 years old (149 million) is stunted (3). In 2021, the prevalence rates of stunting in children under 5 years old in Ecuador, Nigeria, and Ethiopia were, respectively, 23.2, 36.2, and 39% (4–6). In China, 17.07% of children of 3–5 years old had a high risk of early development deficiency in 2021 (7). Early childhood is a critical period of life development, which has a crucial impact on the development and function of the brain (8); plays an important role in the formation of human intelligence, behavior, and social adaptability (9); and even affects adulthood achievement. A study in the field of economics has shown (10) that the average income in adulthood will drop by 26% in a setting of neglect of the key period of child development. Therefore, improving early childhood development is vital to promoting future social development.

Human development is the product of the interaction between certain congenital conditions and acquired developmental environment (11). During the rapid growth in early childhood, the brain particularly is vulnerable to external factors such as nutritional status, socioeconomic factors, and parent–child relationships (1, 12). Such stimuli directly or indirectly have a positive or negative impact on children (13). In developing countries, due to long-term living in poverty, many children experience chronic diseases such as malnutrition and infection in their infancy and toddlerhood. Even if they survive, they easily suffer from neurodevelopmental defects (14). It has been confirmed that in low-income and middle-income environments, high-quality diets in childhood are positively correlated with the cognitive development scores of 24-month-old children, and chronic diseases are negatively correlated with cognitive development (15). Meanwhile, malnutrition affects gastrointestinal function. In a resource-poor environment, many children suffer from impaired intestinal function and environmental enteric dysfunction (16). This has been confirmed as an important cause of developmental delay in children (17). It is worth noting that due to economic development and urbanization transformation, many developing countries are experiencing the double burden of malnutrition and nutrition transition. The increase rate of overweight and obesity is much greater than the decrease rate of malnutrition (18), which will bring new challenges to national policy makers and health professionals. Finally, early education is an important factor in early childhood development (1). In economically developed countries and regions, early childhood education resources are abundant, and more families send their children to professional institutions as early as possible to receive early education and develop their potential (19). On the contrary, 25% of children under the age of 5 years old in developing countries live in extreme poverty (1). Poverty and other factors make early childhood education opportunities in professional institutions very limited. All these have increased the risk of early developmental delay in children in developing countries to a certain extent.

Parenting mainly refers to the care provided by the family for children's health promotion, nutritional needs, response, emotional support, developmental stimulation, etc. (1). Based on the Ecological Systems Theory (20), the parenting environment is the main influencing factor of children's development, which is a nested ecosystem with a core of children. The inner micro-system, i.e., home, has the most direct and significant impact on children development. The home parenting environment includes parental quality, family structure, parent-child response, family resource availability, learning conditions, etc. (1, 21). Family structure, parenting styles, and family relationships are closely related to child development (21). Supporting parents and other child caregivers and improving parenting quality at the family level can promote child developmental potential (22). A multi-arm, cluster-randomized community effectiveness trial in rural western Kenya also reported that parental intervention could effectively promote the early development of children aged 6–24 months (23).

Cognitive and psychomotor development are important indicators of early childhood development (1), including thinking ability, attention, problem solving ability, memory, fine motor, gross motor, and other abilities that help children understand the world (24). In recent decades, the number of studies concerning family environment and child development, especially cognitive development, have gradually increased, but most of them (25, 26) have focused on the impact of general factors such as family economic status, parent's educational level, and parenting methods on child development. Moreover, some studies, which evaluate home environment as a complete system and focus on child development, have small sample sizes; are of single-center design; and do not involve cultural background, socioeconomic differences, and other issues, finally leading to controversial results. For example, Ribe et al. (27) showed that there was no statistically significant relationship between family parenting and the early development of the children of 15 months old. However, Mccormick et al. (28) showed that there was a positive correlation between family parenting and the early childhood development.

Herein, we conducted a meta-analysis to evaluate the overall effect size of the relationship between family parenting environment and early cognition and psychomotor development in children under 5 years old. The observational studies published in Chinese and English from the 1990 s focusing on relationship between the family parenting environment and the cognitive and psychomotor development of children under 5 years of age were included in this meta-analysis. Our findings may provide a basis for promoting early childhood development and potential development.

## Materials and Methods

### Literature Research

The databases of Pubmed, Embase, The Cochrane Library, CNKI, Weipu, Wanfang databases, and CBMdisc databases were searched from January 1, 1990, to July 31, 2021. Each database was searched by using the following key terms: (cognitive development OR psychomotor development OR neuropsychological development) AND (home environment OR development environment) AND (child OR children OR infant OR infants), limited to English or Chinese language.

### Inclusion and Exclusion Criteria

The inclusion criteria were as follows: (1) Observational study. (2) The study population consisted exclusively of children under 5-years-old. (3) Pearson correlation or Spearman correlation was used to investigate the relationship between home parenting environment and children's cognitive development or psychomotor development, and, the correlation coefficient [Pearson correlation coefficient (r) or Spearman correlation coefficient (rs)] was reported. (4) Home parenting environment and children's cognitive and psychomotor development were assessed by validated and reliable scales.

The exclusion criteria were as follows: (1) Non-observational study. (2) Study population was children over 6 years old. (3) Study population involved children with disorders affecting cognitive and psychomotor development. (4) The correlation coefficients between home parenting environment and children's cognitive development or psychomotor development was not reported; (5) The data were incomplete or the effect size cannot be extracted.

### Data Extraction

Two researchers (Yang Q and Yang JL) conducted independent literature search and data extraction according to the search strategy and inclusion and exclusion criteria. The extracted data included the first author, publication year, research location, sample size, sex ratio, measurement tools, age of children at the time of evaluation, and correlation coefficient (r or rs) between home parenting environment and child development. Finally, the two researchers cross-checked the extracted data.

### Outcome Variables

The outcome variables were defined as relationship between home parenting environment and children's cognitive development or psychomotor development, which was represented by correlation coefficient (r or rs).

### Quality Evaluation

The study quality was evaluated according to the STROBE statement (29). A total of 22 items including six aspects of title, abstract, introduction, methods, results, and discussion were assessed. One item was scored 1 point and there were 22 points in total.

### Statistical Analysis

Review Manager 5.4 was used for meta-analysis. Before merging the effect size, Fisher's Z values and SE values were converted according to the formula. The summary Fisher's Z was obtained based on Fisher's Z values and SE values. Cochran *Q*-test and *I*^2^ test were used for heterogeneity test. If *p* > 0.10 and *I*^2^ <50%, there was no statistical heterogeneity between the studies, and the fixed effects model was used. *p* < 0.10 or *I*^2^ > 50% indicates that there is heterogeneity between the studies, and the random effects model was used. The subgroup analysis was performed according to the age of children and the research area. The funnel plot was used to analyze publication bias, and each included study was eliminated one by one to assess sensitivity. Finally, summary r was calculated according to summary Fisher's Z to determine the correlation of home parenting environment and child development. The value range of *r* ≥ 0.4 was defined as strong correlation; 0.1 < *r* < 0.4 as medium correlation; and *r* ≤ 0.1 as weak correlation (30). The formula was as follows (31):


(1) $$\begin{array}{l} \text{r}=2\text{sin}\left(\text{rs}\frac{\pi }{\;6}\right) \end{array}$$

(2) $$\begin{array}{l} \text{Fishe}{\text{r}}^{\text{′}}\text{s Z}=0.5\times \text{In}\frac{1+\text{r}}{1-\text{r}} \end{array}$$

(3) $$\begin{array}{l} \text{SE}=\sqrt{\frac{1}{\text{n}-3}} \end{array}$$

(4) $$\begin{array}{l} \text{Summary r}=\frac{{\text{e}}^{2\text{Z}}-1}{{\text{e}}^{2\text{Z}}+1} \end{array}$$

Note: *r*, Pearson correlation coefficient; rs, Spearman correlation coefficient; SE, standard error; Z, summary Fisher's Z.

## Results

### Literature Research Results

**Figure 1:**
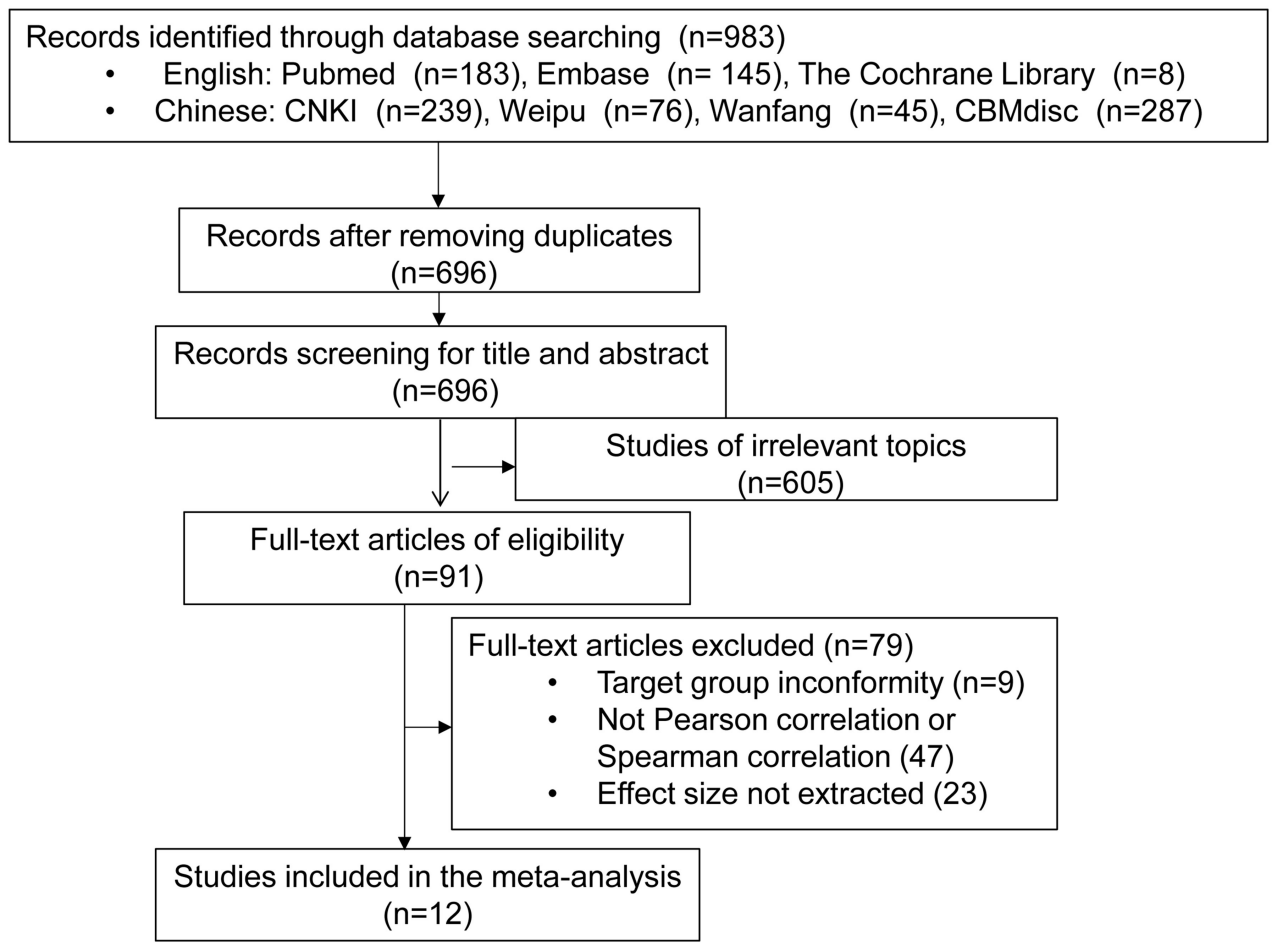
Flow chart of study selection.

The study flowchart is shown in Figure 1. A total of 983 articles were obtained after initial screening, including 336 in English and 647 in Chinese. After excluding duplicate articles, studies of obviously irrelevant topics, studies of inconsistent outcome indicators, or studies without extractable effect size, 12 articles (19, 32–42) were finally included in the meta-analysis. Among them, there were 11 articles in English (19, 32–38, 40–42) and one article in Chinese (39). One article (32) divided the subjects into the 0–17-month-old group and the 18–30 month old group, and investigated the relationship between the early development of children and home parenting environment. Thus, the two groups of data were extracted independently. The study subjects came from eight countries including China, South Africa, the United States, Brazil, Spain, Norway, India, and Bangladesh. One group of the study subjects was Mexican-American (36). The basic characteristics and literature quality of all studies are shown in Table 1. The quality score of the included studies ranged from 10 to 19 points, and the quality scores of three studies were less than half of the total score (≤11 points), which were rated as low quality in this study (32, 33, 39).

**Table 1:** Basic characteristics and quality evaluation of the included studies.

**Included studies**	**Location**	**Cases**	**Male/female**	**Home environment (evaluation tools/age)**	**Children development (evaluation tools/age)**		**Quality evaluation**
					**Cognitive development**	**Psychomotor development**	
Nampijja et al. (41)	America	146	Unknown	HOME/5 y	Bdpv/5 y	–	15
Barreto et al. (40)	Spain	295	145/150	HES/2 y	MSCA/4 y	–	17
Ding et al. (39)	China	188	97/91	CNEQ/12 m	–	NDIC (0–6 y)/2 y	11
Miquelote et al. (19)	Brazil	32	16/16	AHEMD-IS/15 m	Bayley-III/15 m	Bayley-III/15 m	15
Oliveira et al. (38)	Brazil	23	9/14	HOME/5–6 y	WISC III/5–6 y	–	16
Wu et al. (42)	Taiwan China	1,630	900/730	HOME/3 y	TBCS-36/3 y	TBCS-36/3 y	14
Hamadani et al. (37)	Bangladesh	797	Unknown	HOME/18 m	Bayley-III/18 m	Bayley-III/18 m	14
Black et al. (35)	India	162	Unknown	HOME/10 m	Bayley/10 m	Bayley/10 m	19
Kolobe (36)	America (Mexica American)	62	29/33	HOME/12 m	Bayley-III/12 m	Bayley-III/12 m	14
Health (34)	America	260	Unknown	HOME/24 m	Bayley-III/24 m	–	17
Andersson et al. (33)	Norway	142	66/76	HSQ/13 m	FTII/7 m	–	11
Richter and Grieve (32)	South Africa	183	98/85	HSQ/2–17 m	Bayley/2–17 m	Bayley/2–17 m	10
Richter and Grieve (32)	South Africa	122	64/58	HSQ/18–30 m	Bayley/18–30 m	Bayley/18–30 m	10

### Early Development of Children Under 5 Years of Age Is Moderately Correlated With Home Parenting Environment

A total of 12 independent samples in 11 studies (19, 32–38, 40–42) reported the correlation coefficients between home parenting environment and cognitive development in children. The results of meta-analysis under random-effects model (*p* = 0.08, *I*^2^ = 40%) showed that the summary Fisher's Z value was 0.32 (95% CI: 0.27–0.37) (*p* < 0.001). The converted summary *r* value was 0.31. This indicates that cognitive development of children under 5 years old is moderately correlated with the home parenting environment.

A total of eight independent samples in seven studies (19, 32, 35–37, 39, 42) reported the correlation coefficients between home parenting environment and psychomotor development in children. The results of meta-analysis under fixed-effects model (*p* = 0.46, *I*^2^ = 0%) showed that the summary Fisher's Z value was 0.21 (95% CI: 0.17–0.24) (*p* < 0.001) and that the summary r value was 0.21. This indicates that psychomotor development of children under 5 years old is moderately correlated with the home parenting environment.

### Subgroup Analysis

The age of children under development assessment and the national economic level may have a certain impact on the relationship between the home parenting environment and child development. Thus, the included studies were divided into groups of 0–17 months and over 18 months according to the age of children under development assessment. According to economic level, the included studies were divided into a group of developed countries and that of developing countries.

**Figure 2:**
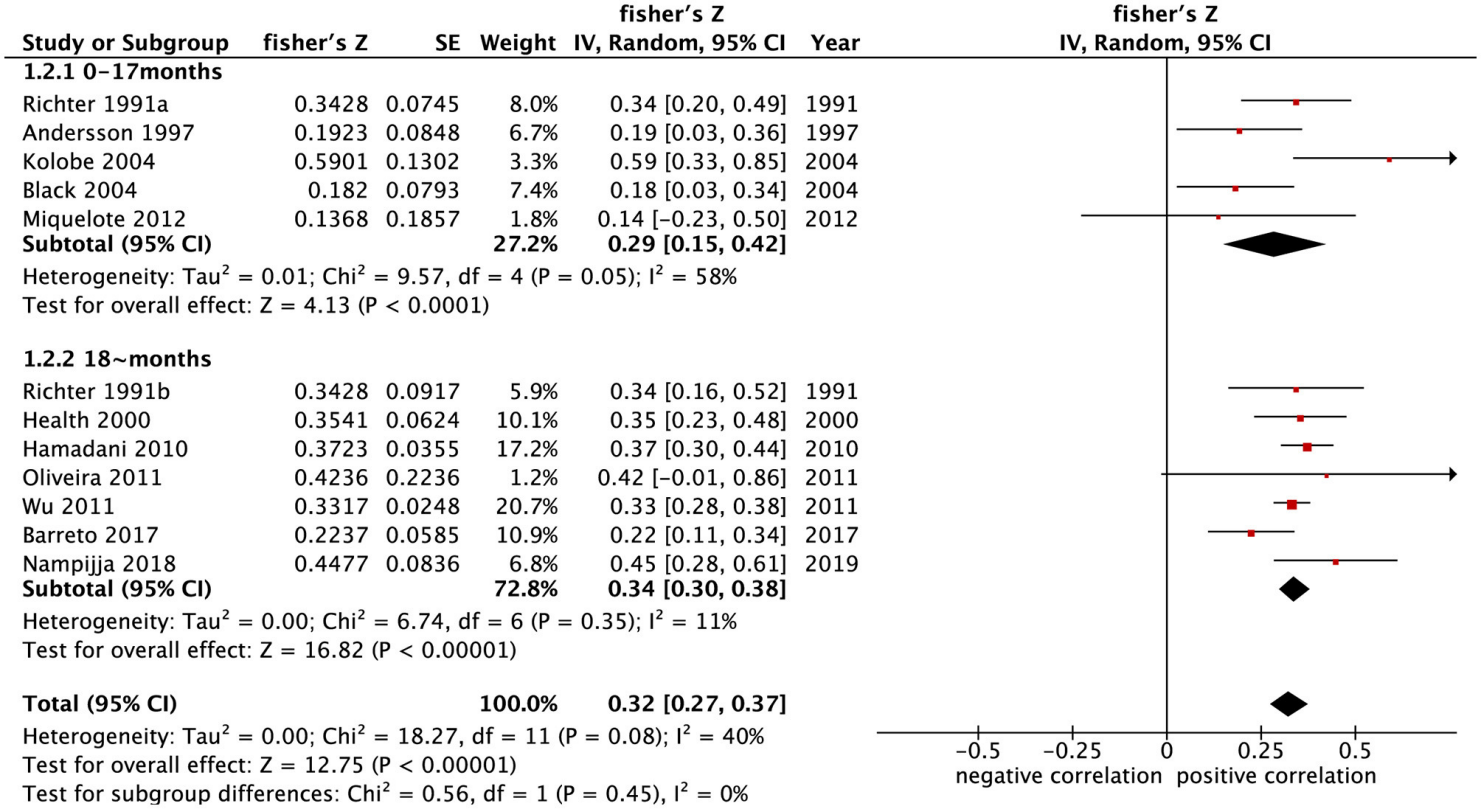
Forest map of the relationship between home parenting environment and cognitive development in children of different age groups.

**Figure 3:**
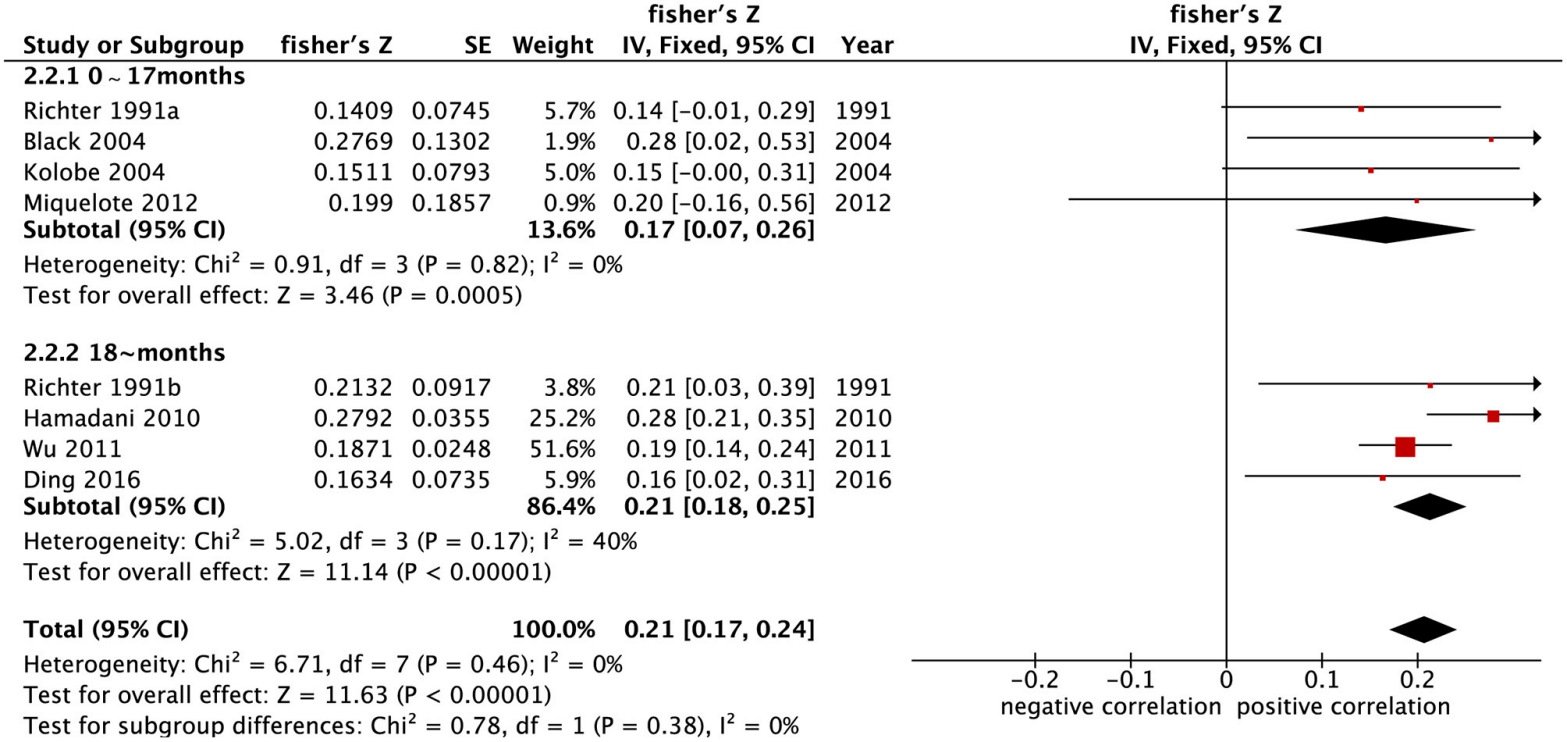
Forest map of the relationship between home parenting environment and psychomotor development in children of different age groups.

The results of the meta-analysis grouped by age of children under development assessment showed that the correlation coefficients combined with the summary Fisher's *Z* value between home parenting environment and cognitive and psychomotor development in the 0–17 months group were [0.29 (95% CI: 0.15–0.42)] and [0.17 (95% CI: 0.07–0.26), *p* < 0.001] (Figures 2, 3), respectively. The correlation coefficients combined with summary Fisher's *Z* values of home parenting environment and cognitive development and psychomotor development in over 18 months group were [0.34 (95% CI: 0.30–0.38), *p* < 0.001] and [0.21 (95% CI: 0.18–0.25), *p* < 0.001] (Figures 2, 3), respectively. The converted summary r values were 0.28, 0.17, 0.33, and 0.21, respectively. From the value of the summary correlation coefficient, the correlation between early childhood development and home parenting environment after 18 months may be stronger than before 17 months.

**Figure 4:**
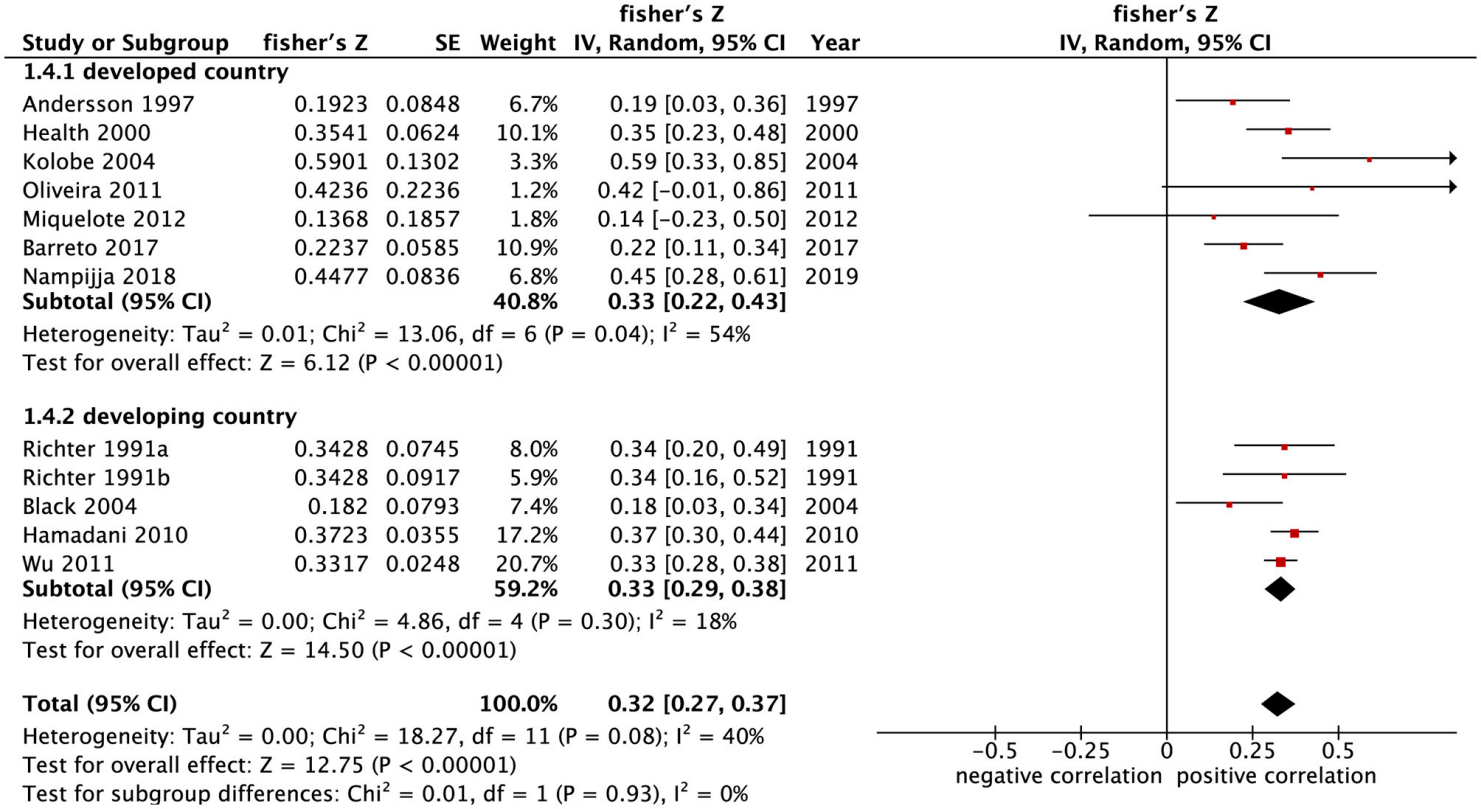
Forest map of the relationship between home parenting environment and cognitive development in developing and developed countries.

Since there were only two developing countries that evaluated the relationship between home parenting environment and psychomotor development, subgroup analysis was not performed on psychomotor development. However, the studies evaluating the relationship between home parenting environment and cognitive development were subjected to subgroup analysis. The results showed that the correlation coefficients combined with summary Fisher's *Z* values of home parenting environment and cognitive development in developing and developed countries were [0.33 (95% CI: 0.29–0.38), *p* < 0.001], and [0.33 (95% CI: 0.22–0.43), *p* < 0.001] (Figure 4). The converted summary *r* value between home parenting environment and cognitive development was both 0.32 in developing and developed countries, suggesting that the correlation between home parenting environment and cognitive development is the same between countries with different economic levels.

### Publication Bias

**Figure 5:**
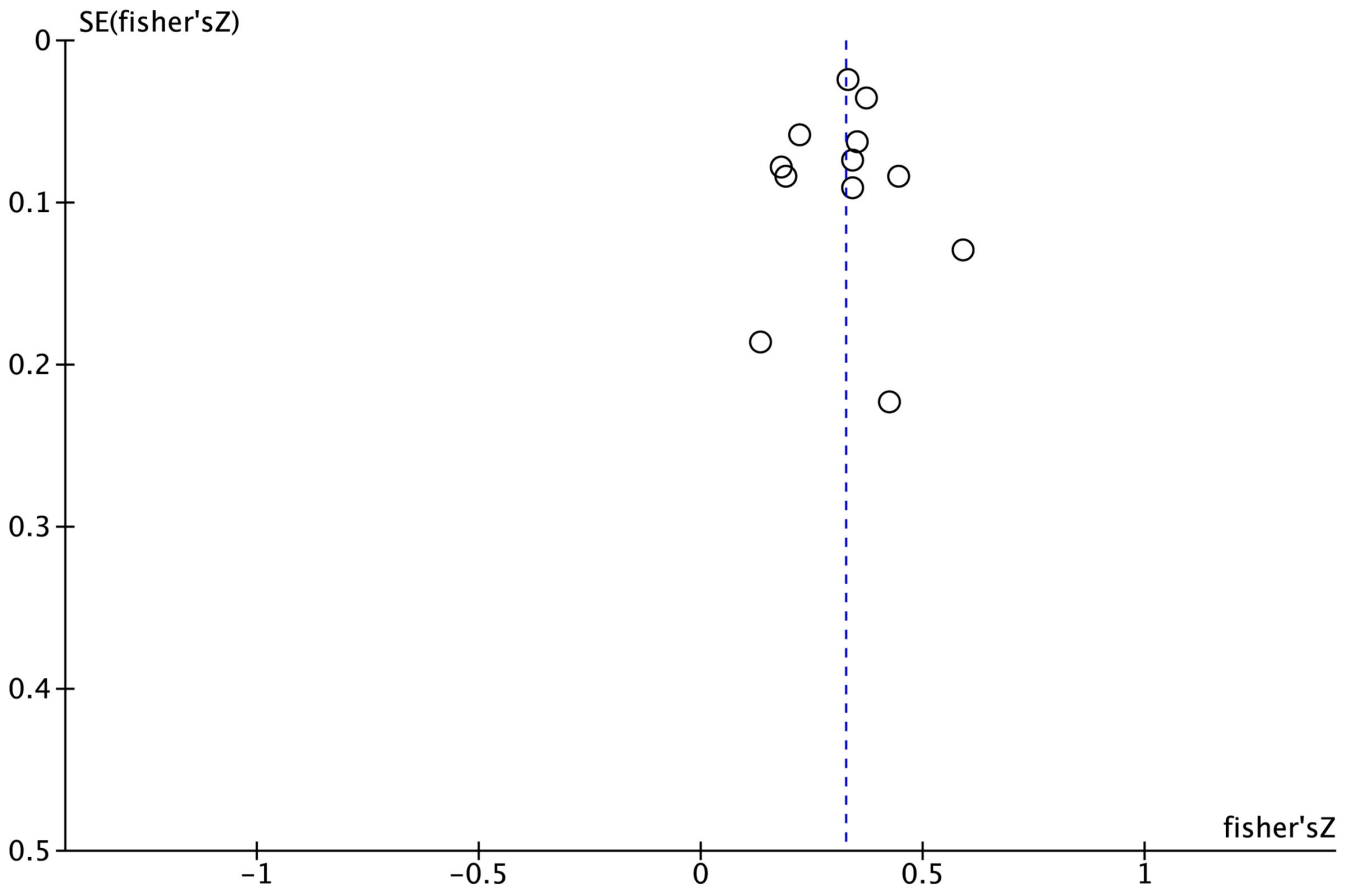
Funnel diagram of relationship between home parenting environment and cognitive development.

Funnel plot was used to analyze the bias of 11 enrolled studies reporting the relationship between home parenting environment and cognitive development. The results were slightly asymmetrical, and there may be a certain degree of publication bias (Figure 5).

### Sensitivity Analysis

The method of eliminating each study one by one was used to observe the influence of a single study on the total combined effect size, and the results were not significantly different from the total combined effect size, suggesting that the study was stable. The results are shown in Table 2.

**Table 2:** Sensitivity analysis of relationship between home parenting environment and children's cognitive development (excluding individual studies one by one).

**Deleted studies**	**Heterogeneity analysis**		**Summary Fisher's *Z* (95% CI*)***
	***p***	***I*^**2**^ (%)**	
Nampijja et al. (41)	0.09	38	0.33 (0.30–0.36)
Barreto et al. (40)	0.14	32	0.34 (0.31–0.37)
Miquelote et al. (19)	0.07	42	0.33 (0.30–0.36)
Oliveira et al. (38)	0.05	45	0.33 (0.30–0.36)
Wu et al. (42)	0.05	45	0.33 (0.29–0.37)
Hamadani et al. (37)	0.09	39	0.32 (0.28–0.35)
Black et al. (35)	0.15	32	0.34 (0.30–0.37)
Kolobe (36)	0.16	30	0.33 (0.29–0.36)
Health (34)	0.05	45	0.33 (0.30–0.36)
Andersson et al. (33)	0.11	36	0.34 (0.30–0.37)
Richter and Grieve (32)	0.05	45	0.33 (0.30–0.36)
Richter and Grieve (32)	0.05	45	0.33 (0.30–0.36)

## Discussion

The development of cognition and movement in infants and young children is very rapid and has strong plasticity, which is known as “golden moment” of neuropsychological development. Among the three major living environments of children (natural environment, social environment, and family environment), the home parenting environment is the most exposed one in early childhood surroundings. This meta-analysis showed that the home parenting environment was moderately positively correlated with the cognitive development of children under 5 years old (*r* = 0.31). Previous studies have also shown that a high-quality home parenting environment has a continuous positive effect on the cognitive development of children under 5 years old (28), which may be because the high-quality home parenting environment can provide children with safe environment, learning support, emotional and verbal responsivity (15), and sufficient stimulation (43) that are conducive to children's cognitive development. Meanwhile, the reasoning ability and depressive symptoms of the mothers, as relevant factors of home parenting environment, also may have an impact on children's early development; however, the negative roles of illness, infection, and poor infant feeding practices will increase the risk of constrained cognitive development in settings with less promotion of development (15). We also found that the home parenting environment was moderately positively correlated with the psychomotor development of children under 5 years old (*r* = 0.21), which is consistent with the results by White-Traut et al. (44). Therefore, improving the home parenting environment in early childhood and providing positive stimulation is conducive to early childhood cognitive and psychomotor development (45, 46).

In order to further explore the factors affecting the relationship between home parenting environment and child development, subgroup analysis was performed in terms of the age of child development assessment and the survey area. Subgroup analysis showed that the home parenting environment was positively correlated with the cognitive development and psychomotor development of children under 17 months and over 18 months. From the perspective of the correlation coefficient, the relationship between the home parenting environment and the development of children over 18 months was stronger than that of children under 17 months [(*r* = 0.33, *r* = 0.21) vs. (*r* = 0.28, *r* = 0.17)]. Miquelote et al. (19) found that the correlation between home parenting environment and child development was stronger at 15 months of age than at 9 months of age. McCormick et al. (15) reported that the home parenting environment at 24 months and 60 months of age could better distinguish the cognitive development trajectory than that at 6 months of age. The results of this meta-analysis are consistent with their results, suggesting that the positive impact of home parenting environment on child development gradually increases with age and that the home parenting environment has continuous effect on the development of children (44). The subgroup analysis also showed that the combined correlation coefficient between home parenting environment and cognitive development was both 0.32 in developing and developed countries, indicating that this correlation of home parenting environment and cognitive development is the same in countries with different economic levels.

There were some limitations in this study. First, papers in languages other than Chinese and English and those using regression analysis to investigate the correlation between home parenting environment and child development were excluded. Second, the funnel plot showed that there may be a certain publication bias, which may have a certain impact on the final effect. Third, this study did not include children over 5 years old and could not provide evidence for the long-term effects of the home parenting environment on children's cognitive and psychomotor development. Finally, the quality of the included papers was mostly between 10 and 19 points, of which the quality scores of three studies were less than half of the total score. Almost all the studies lacked a reasonable description of the research samples, which may affect the scientificity of conclusions. Future studies with more high-quality articles are warranted to investigate the long-term impact of the home parenting environment on the development of children.

In conclusion, the home parenting environment was positively correlated with the cognitive and psychomotor development of children under 5 years old. The older the age, the stronger the correlation, indicating that the home parenting environment may have a continuous positive impact on the development of children. The early development potential of children in the family environment of high quality is relatively better. These results indicate that the main caregivers of children should not only provide children with reasonable dietary nutrition and economic security, but also enhance parent-child interactions, provide appropriate support and stimulation, and create a family environment conducive to the development of children.

## Data Availability Statement

The original contributions presented in the study are included in the article/supplementary material, further inquiries can be directed to the corresponding author/s.

## Author Contributions

LY and QY designed the study. QY and JY collected the data and searched the literatures. QY and LZ analyzed and interpreted the data. LZ and WS prepared the study. QY collected the funds and wrote the paper. LY revised the paper. All authors contributed to the article and approved the submitted version.

## Funding

This study was funded by Scientific Research Project of Hunan Provincial Health Commission, China (No. 20200214).

## Conflict of Interest

The authors declare that the research was conducted in the absence of any commercial or financial relationships that could be construed as a potential conflict of interest.

## Publisher's Note

All claims expressed in this article are solely those of the authors and do not necessarily represent those of their affiliated organizations, or those of the publisher, the editors and the reviewers. Any product that may be evaluated in this article, or claim that may be made by its manufacturer, is not guaranteed or endorsed by the publisher.

## References

[1] Black MM Walker SP Fernald LCH Andersen CT Digirolamo AM Lu C . Early childhood development coming of age: science through the life course. Lancet. (2017) 389:77–90. 10.1016/S0140-6736(16)31389-7 27717614 PMC5884058

[2] Grantham-Mcgregor S Cheung YB Cueto S Glewwe P Richter L Strupp B . Developmental potential in the first 5 years for children in developing countries. Lancet. (2007) 369:60–70. 10.1016/S0140-6736(07)60032-4 17208643 PMC2270351

[3] Development Initiatives. Global Nutrition Report: Introduction: Towards Global Nutrition Equity. (2020). Available online at: https://globalnutritionreport.org/reports/2020-global-nutrition-report/introduction-towards-global-nutrition-equity/ (accessed July 31, 2021).

[4] Hajri T Angamarca-Armijos V Caceres L. Prevalence of stunting and obesity in ecuador: a systematic review. Public Health Nutr. (2021) 24:2259–72. 10.1017/S1368980020002049 32723419 PMC10195486

[5] Amaha ND Woldeamanuel BT. Maternal factors associated with moderate and severe stunting in ethiopian children: analysis of some environmental factors based on 2016 demographic health survey. Nutr J. (2021) 20:18. 10.1186/s12937-021-00677-6 33639943 PMC7916293

[6] Tesfaw LM Fenta HM. Multivariate logistic regression analysis on the association between anthropometric indicators of under-five children in Nigeria: NDHS 2018. BMC Pediatr. (2021) 21:193. 10.1186/s12887-021-02657-5 33888079 PMC8061068

[7] Gao Y Zhang Y Zhao J Shan W Wang X Zhang Z . Association between child care environment and childhood early development. Chin J Pediatr. (2021) 59:175–80. 10.3760/cma.j.cn112140-20200730-00768 33657690

[8] Kiser B. Early child development: body of knowledge. Nature. (2015) 523:286–9. 10.1038/523286a 26178952

[9] Ruys CA Hollanders JJ Broring T Van Schie PEM Van Der Pal SM Van De Lagemaat M . Early-life growth of preterm infants and its impact on neurodevelopment. Pediatr Res. (2019) 85:283–92. 10.1038/s41390-018-0139-0 30140070

[10] Richter LM Daelmans B Lombardi J Heymann J Boo FL Behrman JR . Investing in the foundation of sustainable development: pathways to scale up for early childhood development. Lancet. (2017) 389:103–18. 10.1016/S0140-6736(16)31698-1 27717610 PMC5880532

[11] Weaver IC. Integrating early life experience, gene expression, brain development, and emergent phenotypes: unraveling the thread of nature via nurture. Adv Genet. (2014) 86:277–307. 10.1016/B978-0-12-800222-3.00011-5 25172353

[12] Eves R Mendonca M Bartmann P Wolke D. Small for gestational age-cognitive performance from infancy to adulthood: an observational study. BJOG. (2020) 127:1598–606. 10.1111/1471-0528.16341 32479707

[13] Degeilh F Beauchamp MH Leblanc E Daneault V Bernier A. Socioeconomic status in infancy and the developing brain: functional connectivity of the hippocampus and amygdala. Dev Neurosci. (2019) 41:327–40. 10.1159/000507616 32516794

[14] Galler JR Koethe JR Yolken RH. Neurodevelopment: the impact of nutrition and inflammation during adolescence in low-resource settings. Pediatrics. (2017) 139:S72–84. 10.1542/peds.2016-2828I 28562250 PMC5374755

[15] McCormick BJJ Caulfield LE Richard SA Pendergast L Seidman JC Maphula A . Early life experiences and trajectories of cognitive development. Pediatrics. (2020) 146:e20193660. 10.1542/peds.2019-3660 32817437 PMC7461241

[16] Selimoglu MA Kansu A Aydogdu S Sarioglu AA Erdogan S Dalgic B . Nutritional support in malnourished children with compromised gastrointestinal function: utility of peptide-based enteral therapy. Front Pediatr. (2021) 9:610275. 10.3389/fped.2021.610275 34164352 PMC8215107

[17] Iqbal NT Syed S Sadiq K Khan MN Iqbal J Ma JZ . Study of environmental enteropathy and malnutrition (SEEM) in Pakistan: protocols for biopsy based biomarker discovery and validation. BMC Pediatr. (2019) 19:247. 10.1186/s12887-019-1564-x 31331393 PMC6643315

[18] Mbogori T Kimmel K Zhang M Kandiah J Wang Y. Nutrition transition and double burden of malnutrition in Africa: a case study of four selected countries with different social economic development. AIMS Public Health. (2020) 7:425–39. 10.3934/publichealth.2020035 32968668 PMC7505783

[19] Miquelote AF Santos DC Cacola PM Montebelo MI Gabbard C. Effect of the home environment on motor and cognitive behavior of infants. Infant Behav Dev. (2012) 35:329–34. 10.1016/j.infbeh.2012.02.002 22721733

[20] Buser JM Boyd CJ Moyer CA Ngoma-Hazemba A Zulu D Mtenje JT . Operationalization of the ecological systems theory to guide the study of cultural practices and beliefs of newborn care in rural zambia. J Transcult Nurs. (2020) 31:582–90. 10.1177/1043659620921224 32406802

[21] Orri M Cote SM Tremblay RE Doyle O. Impact of an early childhood intervention on the home environment, and subsequent effects on child cognitive and emotional development: a secondary analysis. PLoS ONE. (2019) 14:e0219133. 10.1371/journal.pone.0219133 31269050 PMC6608972

[22] Britto PR Lye SJ Proulx K Yousafzai AK Matthews SG Vaivada T . Nurturing care: promoting early childhood development. Lancet. (2017) 389:91–102. 10.1016/S0140-6736(16)31390-3 27717615

[23] Luoto JE Lopez Garcia I Aboud FE Singla DR Fernald LCH Pitchik HO . Group-based parenting interventions to promote child development in rural Kenya: a multi-arm, cluster-randomised community effectiveness trial. Lancet Glob Health. (2021) 9:e309–19. 10.1016/S2214-109X(20)30469-1 33341153 PMC8054650

[24] Alam MA Richard SA Fahim SM Mahfuz M Nahar B Das S . Impact of early-onset persistent stunting on cognitive development at 5 years of age: results from a multi-country cohort study. PLoS ONE. (2020) 15:e0227839. 10.1371/journal.pone.0227839 31978156 PMC6980491

[25] Firk C Konrad K Herpertz-Dahlmann B Scharke W Dahmen B. Cognitive development in children of adolescent mothers: the impact of socioeconomic risk and maternal sensitivity. Infant Behav Dev. (2018) 50:238–46. 10.1016/j.infbeh.2018.02.002 29448186

[26] Neel MLM Stark AR Maitre NL. Parenting style impacts cognitive and behavioural outcomes of former preterm infants: a systematic review. Child Care Health Dev. (2018) 44:507–15. 10.1111/cch.12561 29575031 PMC6005730

[27] Ribe IG Svensen E Lyngmo BA Mduma E Hinderaker SG. Determinants of early child development in rural Tanzania. Child Adolesc Psychiatry Ment Health. (2018) 12:18. 10.1186/s13034-018-0224-5 29568326 PMC5859781

[28] Mccormick BJJ Richard SA Caulfield LE Pendergast LL Seidman JC Koshy B . Early life child micronutrient status, maternal reasoning, and a nurturing household environment have persistent influences on child cognitive development at age 5 years: results from MAL-ED. J Nutr. (2019) 149:1460–9. 10.1093/jn/nxz055 31162601 PMC6686051

[29] Von Elm E Altman DG Egger M Pocock SJ Gotzsche PC Vandenbroucke JP . The strengthening the reporting of observational studies in epidemiology (STROBE) statement: guidelines for reporting observational studies. Int J Surg. (2014) 12:1495–9. 10.1016/j.ijsu.2014.07.013 25046131

[30] Lipsy MW Wilson DB. Practical Meta-Analysis. California: Sage (2000).

[31] Borenstein M Hedges L Higgins J. Introduction to Meta-Analysis. New York, NY: Wiley (2009).

[32] Richter LM Grieve KW. Home environment and cognitive development of black infants in impoverished South African families. Infant Mental Health J. (1991) 12:88–102. 10.1002/1097-0355(199122)12:2<88::AID-IMHJ2280120202>3.0.CO;2-Q

[33] Andersson HW Gotlieb SJ Nelson KG. Home environment and cognitive abilities in infants born small-for-gestational-age. Acta Obstet Gynecol Scand Suppl. (1997) 165:82–6. 9219463

[34] Health NIOC Network HDECCR. The relation of child care to cognitive and language development. Child Dev. (2000) 71:960–80. 10.1111/1467-8624.00202 11016559

[35] Black MM Sazawal S Black RE Khosla S Kumar J Menon V. Cognitive and motor development among small-for-gestational-age infants: impact of zinc supplementation, birth weight, caregiving practices. Pediatrics. (2004) 113:1297–305. 10.1542/peds.113.5.1297 15121945 PMC3140639

[36] Kolobe TH. Childrearing practices and developmental expectations for mexican-American mothers and the developmental status of their infants. Phys Ther. (2004) 84:439–53. 10.1093/ptj/84.5.439 15113277

[37] Hamadani JD Tofail F Hilaly A Huda SN Engle P Grantham-Mcgregor SM. Use of family care indicators and their relationship with child development in Bangladesh. J Health Popul Nutr. (2010) 28:23–33. 10.3329/jhpn.v28i1.4520 20214083 PMC2975843

[38] Oliveira GE Magalhães LC Salmela LF. Relationship between very low birth weight, environmental factors, and motor and cognitive development of children of 5 and 6 years old. Rev Bras Fisioter. (2011) 15:138–45. 10.1590/S1413-35552011000200009 21789364

[39] Ding L He S Zhou Q Xu X Tang J Zhang Y. Prospective study of home nurture environment on early childhood developmental quotients and social emotional development. Chin J Child Health Care. (2016) 24:910–2. 10.11852/zgetbjzz2016-24-09-05

[40] Barreto FB Sánchez De Miguel M Ibarluzea J Andiarena A Arranz E. Family context and cognitive development in early childhood: a longitudinal study. Intelligence. (2017) 65:11–22. 10.1016/j.intell.2017.09.006

[41] Nampijja M Kizindo R Apule B Lule S Muhangi L Titman A . The role of the home environment in neurocognitive development of children living in extreme poverty and with frequent illnesses: a cross-sectional study. Wellc Open Res. (2018) 3:152. 10.12688/wellcomeopenres.14702.1 30687794 PMC6338129

[42] Wu JC Chiang TL Bradley RH. Adaptation and validation of the HOME-SF as a caregiver-report home environment measure for use in the Taiwan birth cohort study (TBCS). Early Child Dev Care. (2011) 7:949–65. 10.1080/03004430.2010.504881

[43] Nurliyana AR Mohd Shariff Z Mohd Taib MN Gan WY Tan KA. Early growth and home environment are associated with cognitive development in the first year of life of Malaysian infants. Early Hum Dev. (2019) 140:104890. 10.1016/j.earlhumdev.2019.104890 31655334

[44] White-Traut RC Rankin KM Yoder J Zawacki L Campbell S Kavanaugh K . Relationship between mother-infant mutual dyadic responsiveness and premature infant development as measured by the Bayley III at 6 weeks corrected age. Early Hum Dev. (2018) 121:21–26. 10.1016/j.earlhumdev.2018.04.018 29730131 PMC8656267

[45] Pereira KR Valentini NC Saccani R. Brazilian infant motor and cognitive development: longitudinal influence of risk factors. Pediatr Int. (2016) 58:1297–306. 10.1111/ped.13021 27084989

[46] Obradovi,ć J Yousafzai AK Finch JE Rasheed MA. Maternal scaffolding and home stimulation: key mediators of early intervention effects on children's cognitive development. Dev Psychol. (2016) 52:1409–21. 10.1037/dev0000182 27505702

